# Etude de la prévalence des infections nosocomiales et des facteurs associes dans les deux hopitaux universitaires de Lubumbashi, République Démocratique du Congo: cas des Cliniques Universitaires de Lubumbashi et l’Hôpital Janson Sendwe

**DOI:** 10.11604/pamj.2016.24.275.7626

**Published:** 2016-07-27

**Authors:** Danny Kasongo Kakupa, Prosper Kalenga Muenze, Baudouin Byl, Michèle Dramaix Wilmet

**Affiliations:** 1 Ecole de Santé Publique, Université Libre de Bruxelles, Belgique; 2 Faculté de Médecine, Université de Lubumbashi, Lubumbashi, République Démocratique du Congo

**Keywords:** Infections nosocomiales, prévalence, patients, hôpital, RD Congo, Infections nosocomiales, prévalence, hôpitaux

## Abstract

**Introduction:**

Estimer la prévalence « un jour donné » des infections nosocomiales et déterminer leurs facteurs associés, ensuite estimer la prévalence des micro-organismes responsables des infections nosocomiales de Lubumbashi, République Démocratique du Congo.

**Méthodes:**

Une étude transversale descriptive a été menée dans les deux hôpitaux de Lubumbashi au sein de cinq services d’hospitalisation (Chirurgie, Gynéco-Obstétrique, Médecine interne, Pédiatrie et Réanimation). L’échantillon était constitué de 171 patients hospitalisés et qui ont été interrogés à l’aide d’un questionnaire standardisé. La fiche médicale nous a permit de connaitre le type d’antibiotique administré au patient 48 heures après d’admission. Notre étude s’est déroulée durant le mois de février 2010 dans le cadre de la première enquête locale de prévalence des infections nosocomiales.

**Résultats:**

Notre étude a permis de recenser 59 patients atteints d’une infection nosocomiale. La prévalence globale est de 34,5% (dont 17,0% pour une infection nosocomiale acquise et 17,5% pour une infection importée). L’infection nosocomiale a été définie selon l’Organisation Mondiale de la Santé comme toute infection acquise pendant un séjour à l’hôpital et qui n’était ni présente ni en incubation au moment de l’admission du patient. Les facteurs de risque suivants ont été associés aux infections nosocomiales acquises: durée d’hospitalisation (les patients admis en long séjour, séjour de plus de sept jours d’hospitalisation avaient un risque plus élevé que ceux admis en séjour court, séjour inférieur ou égal à sept jours d’hospitalisation (Ratio de prévalence: RP =3,6 [IC a 95% 1,4-8,9]). Parmi les infections nosocomiales, les infections du site opératoire étaient les plus fréquentes (27,1%), suivies des infections pulmonaires (22,0%) et des infections urinaires (17,0%). L’examen microbiologique a permis de mettre en évidence cinq germes responsables d’une infection nosocomiale chez les patients infectés : Escherichia coli (11,9%), Staphylococcus aureus (6,8%), Pseudomonas aeruginosa (5,1%), Shigella spp (5,1%) et Salmonela typhi (1,7%). L’examen microbiologique n’a été réalisé que dans 31,0 % (n=59). La cefotaxime, céphalosporine de 3ème génération était l’antibiotique le plus prescrit (37,9%), suivi de l’amoxicilline (19,6%) et l’ampicilline (16,3%) en monothérapie. La bi et la trithérapie ont été également prescrites. La voie parentérale était la plus utilisée pour administrer un anti-infectieux. La prévalence d’infections nosocomiales différait significativement entre les deux hôpitaux universitaires ; la prévalence d’une infection nosocomiale acquise est de 22,2% aux Cliniques Universitaires de Lubumbashi et 13,1% à l’hôpital Sendwe.

**Conclusion:**

Dans notre travail, la prévalence globale des infections nosocomiales était de 34,5%. Les infections du site opératoire étaient les plus fréquentes (27,1%). L’Escherichia coli était le germe le plus fréquent soit 11,9%.

## Abstract (en)

**Introduction:**

To estimate the prevalence “on any given day” of nosocomial infections and to determine their associated factors. Then, to estimate the prevalence of microorganisms responsible for nosocomial infections in Lubumbashi, Democratic Republic of Congo.

**Methods:**

A descriptive cross-sectional study was conducted in two hospitals in Lubumbashi in five inpatient units (Surgery, Gynecology and Obstetrics, Internal Medicine, Pediatrics and Recovery). The sample consisted of 171 hospitalized patients who were questioned using a standardized questionnaire. Patient’s medical record allowed us to know the type of antibiotic administered to the patient 48 hours after admission. Our study was conducted in February 2010 as part of the first local prevalence survey on nosocomial infections.

**Results:**

Our study collected data on 59 patients with nosocomial infection. The overall prevalence was 34.5% (17.0% with acquired nosocomial infection and 17.5% with imported infection). According to the World Health Organization, nosocomial infection is a hospital-acquired infection which was not present or incubating at the time of patient admission. The following risk factors have been associated with acquired nosocomial infections: duration of hospitalization (long stay hospital patients, hospital length of stay of more than seven days has a higher risk than shorter length of stay, hospital length of stay of less than or equal to seven days (prevalence ratio: RP =3.6 [IC A 95%.1.4-8.9])). Among nosocomial infections, surgical site infections were the most common (27.1%), followed by lung infections (22.0%) and urinary tract infections (17.0%). Microbiological examination highlighted five germs responsible for nosocomial infection in infected patients: Escherichia coli (11.9%), Staphylococcus aureus (6.8%), Pseudomonas aeruginosa (5.1%), Shigella spp (5.1%) and Salmonella typhimurium (1.7%). Microbiological examination was performed in 31.0% (n = 59). Cefotaxime, third-generation cephalosporin was the most prescribed antibiotic (37.9%), followed by amoxicillin (19.6%) and ampicillin (16.3%) for monotherapy. Dual and triple therapy was also prescribed. Parenteral route was the most used for anti-infective administration. There was a significant difference in the prevalence of nosocomial infections between the two university hospitals; the prevalence of acquired nosocomial infection was 22.2% in University Clinics of Lubumbashi and 13.1% in Sendwe hospital.

**Conclusion:**

In our study, the overall prevalence of nosocomial infections was 34.5%. Surgical site infections were the most common (27,1%). Escherichia coli was the most common germ (11.9%).

## Introduction

Les infections nosocomiales aussi appelées « infections hospitalières » sont définies par l’Organisation Mondiale de la Santé (OMS) comme des infections acquises pendant un séjour à l’hôpital et qui n’étaient ni présentes ni en incubation au moment de l’admission du patient « lorsque la situation précise à l’admission n’est pas connue, un délai d’au moins 48 heures après admission (ou un délai supérieur à la période d’incubation lorsque celle-ci est connue) est communément accepté pour distinguer une infection d’acquisition nosocomiale d’une infection communautaire » [1]. Jusqu’au milieu des années 1980, le risque infectieux nosocomial était sous-estimé. Ce risque a été longtemps négligé en Afrique sub-saharienne, même si la prévalence des infections nosocomiales est plus élevée que dans les pays développés. La prise de conscience de la réalité de ce phénomène, dans un contexte d’amélioration de la qualité des soins, a conduit à ériger la maîtrise du risque infectieux nosocomial en véritable priorité sanitaire [2]. En 2009, l’OMS estimait que 1,4 millions de personnes étaient malades dans le monde de suite d´infections contractées en milieu hospitalier. Dans les pays développés, ces infections touchent 5 à 10 % des patients [3]. La prévalence des infections nosocomiales (IN) est de 4,5 % aux USA, 10,5 % au canada, 6,7 %, en France et 6,2 % en Belgique [3, 4].

En Afrique et dans certains pays en développement, le taux le plus élevé de prévalence de ces infections est estimé à 25,0 % [5]. C’est le cas de l’Albanie en 2009 où la prévalence des IN était de 19,1% ; au Brésil avec 14,0% ; la Tunisie avec 17,8 % ; la Tanzanie avec 14,0 % [3]. En République Démocratique du Congo, en 2011 la prévalence des infections nosocomiales dans les hôpitaux à Kinshasa était estimée à 15,0 % ; le Sénégal avec 10,9%; la Côte d’ivoire avec 12,0 % ; le Bénin avec 10,0 % et le Mali avec 14,0% [6]. Une étude réalisée au Maroc en 2006 a montré un taux d’infections nosocomiales de 17,8 % [7]. La prévalence des IN est nettement plus élevée dans les pays en développement que dans les pays développés. Les infections nosocomiales ne sont pas « le prix à payer » du progrès médical, car elles sont au moins en partie évitables comme l’ont montré certains pays en développant une politique de prévention [8]. Dans les pays développés, les infections nosocomiales constituent 1’une de dix principales causes de mortalité et entre 20 à 30 % de ces infections sont considérées évitables par des méthodes simples et efficaces [5]. C’est le cas de la France, où en 1988 a été créé le Comité de Lutte Contre les Infections Nosocomiales, CLIN en sigle. Il assure la surveillance des infections nosocomiales, rédige des recommandations, forme le personnel, valide les protocoles de soins et participe au contrôle de la prescription des antibiotiques [9].

Par contre, dans les pays en développement, on estime que les infections nosocomiales constituent la troisième cause la plus fréquente de la mortalité et jusqu’à 40 % sont considérées comme évitables [8]. Aujourd’hui, les infections nosocomiales constituent un réel problème en matière de santé publique. Ces infections représentent un frein important au développement médical du fait de leur fréquence sans cesse croissante, de leur gravité et du fait de la multirésistance des germes en cause sans compter l’aspect médicolégal. Elles génèrent un surcoût économique majeur à l’hospitalisation. De cette façon, ces infections contribuent de manière importante à la morbidité et à la mortalité chez des patients hospitalisés [3, 4]. Le présent travail avait pour objectif d’estimer la prévalence des infections nosocomiales et de déterminer les facteurs associés de ces infections, ensuite estimer la prévalence des micro-organismes responsables des infections nosocomiales de Lubumbashi.

## Méthodes

Il s’agit d’une étude transversale descriptive portant sur l’analyse de la prévalence instantanée des infections nosocomiales, avec un seul passage par service, à raison de 1 à 2 service (s) visité (s) par jour (selon la taille des services). Notre étude s’est déroulée durant le mois de février 2010 dans le cadre de la première enquête locale de prévalence de l’infection nosocomiale. Cette étude a été réalisée dans les cinq services d’hospitalisation (Chirurgie, Gynéco-Obstétrique, Médecine interne, Pédiatrie et Réanimation) potentiellement concernés par les infections nosocomiales et la participation des ces établissements sanitaires (ES) étaient volontaires (Cliniques Universitaires de Lubumbashi et l’hôpital provincial de référence J. Sendwe).

La population concernée était composée de l’ensemble des patients présents au moins 48 heures dans le service. Ainsi, ces cinq services d’hospitalisation ont été inclus dans l’enquête à part ceux où le séjour n’excède pas 48 heures (hôpital du jour, laboratoires).

L’échantillon de notre étude est globalement constitué de 171 patients présents au moment de l’enquête et ayant accepté d’être interrogés. 52 patients (23,3 %) étaient non-répondants (refus de réponse, absents au moment de l’enquête, sujets in interrogeables).

Le recueil de données s’est fait par un questionnaire standardisé rempli par un enquêteur formé, un médecin stagiaire (étudiant en dernière année de médecine) supervisé par le médecin du service de l’hôpital. Une pré-enquête a été réalisée 48 heures avant l’enquête. La durée moyenne de l’enquête par malade était de 15 minutes. La fiche de recueil de données contenait les données suivantes : caractéristiques des patients, facteurs de risque, techniques invasives, caractéristiques cliniques et microbiologiques. Les critères de définition d’une infection nosocomiale utilisés étaient basés sur ceux adaptés par l’OMS [1].

## Résultats

### Taux de participation

Aux Cliniques Universitaires de Lubumbashi (CUL), 72 patients ont participé à l’enquête soit un taux de participation de 81,8%. A l’hôpital provincial de référence J. Sendwe, 99 patients ont participé à l’enquête soit un taux de participation de 73,3%. Le taux de participation global dans les deux établissements sanitaires était de 76,7%.

### Description de l’échantillon

#### Distribution de l’âge des patients, en fonction de l’établissement sanitaire

**Figure 1:**
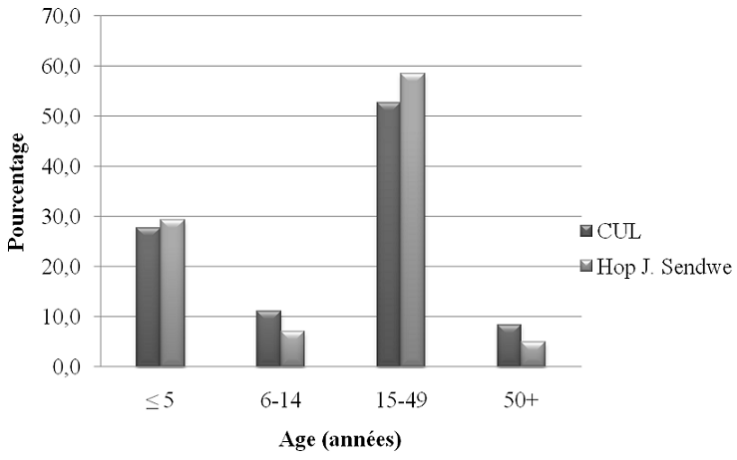
L’âge des patients le jour de l’enquête, par établissement sanitaire

Les sujets âgés de 15 à 49 ans représentaient un peu plus de la moitié de la population hospitalière. L’âge médian des patients le jour de l’enquête était de 24 ans et la durée médiane du séjour hospitalier des patients était de 9 jours. Le sex-ratio (H/F) était de 0,65 (Figure 1, Tableau 1). L’analyse du Tableau 1 montre que dans la distribution des pathologies associées aux infections nosocomiales dans les deux hôpitaux, les proportions des patients avec Broncho-pneumopathie Chronique Obstructive (BPCO), les patients diabétiques et les malnutris différaient statistiquement entre les deux hôpitaux. Cette différence significative était observée aussi pour le service d’hospitalisation

**Tableau 1:** Caractéristiques sociodémographiques et état pathologique du patient, par établissement sanitaire

Facteurs	Total	CUL n (%)	Hop J. Sendwe n (%)	P
**Age (années)**	171	77	99	0,60
≤ 5	28,7	27,8	29,3	
6-14	8,8	11,1	7,1	
15-49	56,1	52,8	58,6	
50+	6,4	8,3	5,0	
**Sexe**	171	72	99	0,57
Masculin	39,2	41,7	37,4	
Féminin	60,8	58,3	62,6	
**BPCO**	151	71	80	0,03
Oui	11,9	5,6	17,5	
Diabète	153	71	82	0,01
Oui	9,2	2,8	14,6	
Malnutrition	171	72	99	0,01
Oui	4,7	0,0	8.1	
**Durée d'hospitalisation**	171	72	99	0,18
Court séjour	42,7	48,6	38,4	
Long séjour	57,3	51,4	61,6	
**Service d'hospitalisation**	169	72	97	0,03+
Chirurgie	26,0	33,3	20,6	
Gynéco-Obstétrique	31,4	26,4	35,1	
Réanimation	4,1	1,4	6,2	
Pédiatrie	23,7	18,1	27,8	
Médecine interne	4,7	20,8	10,3	

+ fichier exact

**Fréquence de prescription des anti-infectieux à usage courant et voie d’administration** En ce qui concerne les anti-infectieux et/ou les antibiotiques, la cefotaxime (céphalosporine de 3^ème^ génération) était l’antibiotique le plus prescrit dans les deux établissements sanitaires (37,9%), venaient ensuite, l’amoxicilline (19,6%) et l’ampicilline (16,3%). Celle-ci a été plus administrée dans les infections nosocomiales acquises que dans les infections importées. La voie parentérale était la plus utilisée pour administrer un anti-infectieux dans les deux hôpitaux et en cas d’infection.

### Prévalence des infections nosocomiales

Le jour de l’enquête dans les deux établissements, le nombre de patients infectés d’une infection nosocomiale était de 59 patients avec un taux de prévalence de 34,5 %. L’analyse du Tableau 2 montre que la prévalence d’infection nosocomiale différait significativement entre les deux hôpitaux. On observait une proportion plus élevée d’infection nosocomiale importée à l’hôpital J. Sendwe.

**Tableau 2:** Prévalence des infections nosocomiales par établissement sanitaire

Prévalence des IN	CUL n=72 (%)	Hop J. Sendwe n=99 (%)	p
Pas d'infection	50 (69,4)	62 (62,6)	
IN importées	6 (8,3)	24 (24,2)	0,015
IN acquises	16 (22,2)	13(13,1)	

Le Tableau 3 montre que les infections du site opératoire étaient dominantes, suivies des infections pulmonaires et des infections urinaires. L’examen microbiologique n’a été réalisé que dans 31,0 % (n=59). Les germes responsables d’une infection nosocomiale chez les patients étaient l’Escherichia coli, le *staphylococcus aureus*, le*pseudomonas aeruginosa*, *shigella spp* et *salmonela typhi*.

#### Prévalence des patients infectés d’une infection nosocomiale acquise

Les patients infectés d’une infection acquise dans les hôpitaux universitaires étaient 29 soit 17,0% (dont 55,2% aux CUL et 44,8% à Sendwe). Seuls la durée d’hospitalisation et le motif d’admission des patients étaient statistiquement associés à l’acquisition d’une infection nosocomiale. Le séjour long (plus de 7 jours d’hospitalisation) était associé significativement à l’acquisition d’une infection nosocomiale : RP (IC95%): 3,6 [1,43-8,92] que le séjour court. Après stratification, l’établissement sanitaire a un effet sur l’association entre l’acquisition d’une infection nosocomiale et la durée d’hospitalisation.

**Tableau 3:** Prévalence des infectés d’une infection nosocomiale par site infectieux et micro-organisme

Facteurs	Total	IN acquise n(%)	IN importée n(%)
**Site infectieux**	**59**	**29**	**30**
Infections pulmonaires	22,0	2 (6,9)	11 (36,7)
Infections urinaires	16,9	8 (27,6)	2 (6,7)
Infections du site opératoire	27,1	9 (31.0)	7 (23,3)
Infections sur cathéter	6,8	2 (6,9)	2 (6,7)
Septicémies	8,5	4 (13,8)	1 (3,3)
Infections non classées	18,6	4 (13,8)	7 (23,3)
**Micro-organismes isolés dans des hémocultures**	**59**	**29**	**30**
*Escherichia coli*	11,9	4 (13,8)	3 (10,0)
*Pseudomonas aeruginosa*	5,1	2 (6,9)	1(3,3)
*Staphylococcus aureus*	6,8	3 (10,3)	1 (3,3)
*Shigella sp*	5,1	2 (6,9)	1 (3,3)
*Salmonela typhi*	1,7	1 (3,4)	0 (0,0)

## Discussion

L’objectif principal de cette étude a été d’estimer la prévalence des patients atteints d’une infection nosocomiale et celle des micro-organismes responsables d’une infection nosocomiale. Pour répondre à cet objectif, une enquête a été menée dans les deux hôpitaux universitaires de Lubumbashi en février 2010. De nombreuses études ont été publiées concernant les infections nosocomiales dans les pays développés contrairement à ceux en voie de développement et plus particulièrement en Afrique.

La comparaison des taux de prévalence rapportés dans notre travail avec les valeurs rapportées dans certains pays développés (USA, Canada, France et Belgique) et en développement (Albanie, Brésil, Tunisie, Maroc, Mali, Tanzanie) est difficile à cause de différences d’ordre méthodologique. Ces différences concernent les critères de définition des infections nosocomiales, le mode de recueil de données, le nombre de sites infectieux investigués, ainsi que le type d’hôpital ou la taille du service étudié.

Les résultats de notre étude ont montré que la prévalence globale des patients infectés d’une IN (acquise et importée) en février 2010 dans les deux ES a été évaluée à 34,5 %. Notre attention s’est focalisée à l’acquisition d’une infection nosocomiale. La prévalence des patients atteints d’une infection nosocomiale acquise était de 17,0%. Ce chiffre est situé dans la fourchette des taux publiés dans la littérature par l’OMS: Mali, 2011: 14,0%, Tanzanie, 2009 :14,8%, Tunisie 17,8% et Maroc 17,8% [3, 7, 8]. Ce chiffre est plus élevé que celui trouvé par Dunia E. et Mwandi A. (15,0%) dans quelques établissements de référence de Kinshasa [6]. Par rapport aux pays développés, ce taux était 3 à 4 fois plus élevé qu’aux USA, 2 à 3 fois plus élevé qu’en France et 3 fois plus élevé qu’en Belgique [6, 8, 10]. L’étude a également montré qu’il y avait une différence significative entre les deux hôpitaux. Ceci peut être expliqué par le fait que l’hôpital J. Sendwe accueille les malades à plus haut risque. En ce qui concerne les sites infectieux, l’infection du site opératoire (27,1%), l’infection pulmonaire (22,0%) et l’infection urinaire (17,0%) étaient les plus fréquentes. Ces trois localisations pourraient être liées à un déficit d’hygiène hospitalière par une insuffisance au niveau de l’entretien du matériel et équipement ou la défaillance du lavage des mains, ou à la durée de la pose de la sonde urinaire. La prédominance de ces trois sites infectieux est cohérente avec les autres enquêtes de prévalence [11]. Notre enquête offre aussi une description de l’écologie bactérienne liée aux infections nosocomiales : *escherichia coli*, *staphylococcus aureus*, *pseudomonas aeruginosa* et *shigella spp*. Cette écologie est similaire à celle décrite dans d’autres enquêtes européennes à l’exception du *shigella spp* [12, 13].

## Conclusion

La prévalence globale d’une infection nosocomiale dans les deux établissements était de 34,5 %. La sonde urinaire et le cathéter vasculaire étaient les principales sources de contamination. Les infections du site opératoire étaient dominantes et les germes responsables d’une infection nosocomiale chez les patients étaient l’*escherichia coli*, le*staphylococcus aureus*, le *pseudomonas aeruginosa*, *shigellaspp* et *salmonelatyphi*. Le transfert des patients infectés d’un autre ES devait se faire dans les meilleures conditions possibles pour être pris correctement en charge dans l’ES d’accueil et éviter une hétéro-infection ou une infection croisée. D’autres facteurs de risque n’ayant pas fait l’objet de notre étude nécessitent une attention particulière. C’est notamment l’asepsie, le lavage des mains et la gestion des déchets hospitaliers. Les plus grands efforts doivent être consacrés à l'éducation des travailleurs de la santé et des patients à l'utilisation correcte des dispositifs invasifs, l’utilisation correcte d’antibiotiques, sur l'importance du lavage des mains dans des conditions aseptiques, du respect de cette consigne dans tout établissement de soins.

### Etat des connaissances actuelles sur le sujet

- En Afrique et dans certains pays en développement, le taux le plus élevé de prévalence de ces infections est estimé à 25,0 %. En République Démocratique du Congo en 2011; la prévalence des infections nosocomiales dans certains hôpitaux de Kinshasa était estimée à 15,0 %;
- Parmi les infections nosocomiales, les infections du site opératoire étaient les plus fréquentes (27,1%), suivies des infections pulmonaires (22,0%) et des infections urinaires (17,0%);
- L’examen microbiologique a permis de mettre en évidence cinq germes responsables d’une infection nosocomiale chez les patients infectés: escherichia coli (11,9%), Staphylococcus aureus (6,8%), Pseudomonas aeruginosa (5,1%), Shigella spp (5,1%) et Salmonela typhi (1,7%). La voie parentérale était la plus utilisée pour administrer un anti-infectieux.

### Contribution de notre étude à la connaissance

- Notre étude ajoute la prévalence d’une infection nosocomiale importée. La prévalence globale est de 34,5% (dont 17,0% pour une infection nosocomiale acquise et 17,5% pour une infection importée);
- Les facteurs de risque suivants ont été associés aux infections nosocomiales acquises: durée d’hospitalisation (les patients admis en long séjour, séjour de plus de sept jours d’hospitalisation avaient un risque plus élevé que ceux admis en séjour court, séjour inférieur ou égal à sept jours d’hospitalisation (Ratio de prévalence: RP =3,6 [IC a 95% 1,4-8,9]);
- La cefotaxime, céphalosporine de 3ème génération était l’antibiotique le plus prescrit (37,9%), suivi de l’amoxicilline (19,6%) et l’ampicilline (16,3%) en monothérapie.
